# Redundancy in Cortical Surface Vessels Supports Persistent Blood Flow

**DOI:** 10.1371/journal.pbio.0040043

**Published:** 2006-01-03

**Authors:** Liza Gross

While examining the brain of a man who had died of a mesenteric tumor, Sir Thomas Willis was shocked to find a nearly totally blocked right carotid artery, the brain's main blood source. In his 1664 book, *Anatomy of the Brain*, the Oxford physician and neuroanatomist reported that given the “influx of blood being denied by this route, it seemed remarkable that this person had not previously died of an apoplexy.” A stroke was averted, Willis explained, because the circular arrangement of the arteries on the base of the brain—later named the circle of Willis—created redundant connections in the circulatory network.

Scientists are still elucidating the details of vascular resilience—and how stroke can result when it is compromised. Much as the circle of Willis protects large-scale blood flow, it's been a matter of conjecture if a similar mechanism ensures microvessel flow to local brain regions. Until recently, scientists have lacked the appropriate methods to test this hypothesis. In a new study, Chris Schaffer, David Kleinfeld, and their physics and medical school colleagues take advantage of cutting-edge techniques in microscopy and laser-induced clotting (photothrombosis) to directly measure the resilience and dynamics of cortical blood flow. By manipulating and monitoring blood flow in the surface vasculature of rat parietal cortex, they show that the arteriole network rapidly reestablishes blood flow following a targeted occlusion to a surface vessel. [Fig pbio-0040043-g001]

**Figure pbio-0040043-g001:**
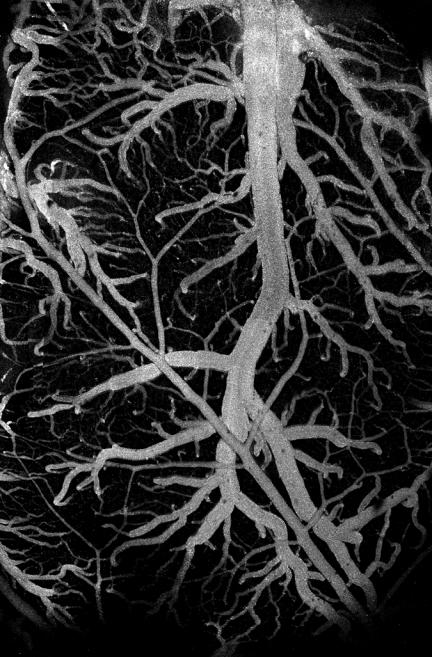
The surface arteriole communicating network in the rat

To analyze flow dynamics among the interconnected arterioles on the cortical surface, Schaffer et al. labeled blood plasma with a dye that allowed them to map the vessel architecture and also measure the flow of individual blood cells, using two-photon microscopy. Candidate vessels were selected for photothrombotic clotting, a technique that uses photosensitive molecules and focal laser beams to produce free radicals and damage the vessel wall, ultimately triggering an occluding blood clot. This enabled the authors to pinpoint a single vessel for occlusion without harming any neighboring vessels. They further used two-photon microscopy to determine the direction and speed of red blood cells by repetitively scanning the axis of each vessel, which allowed them to monitor the pattern of blood flow throughout the surface vascular network in real time.

Schaffer et al. first tested the brain's vascular resilience by inducing targeted photothrombotic clots in an individual surface arteriole upstream of a branch point. Despite the blockage, blood flow was maintained in both downstream branches because of a reversal in the direction of flow through one of the two branches—just one second after occlusion. Of a total 47 clots in 34 rats, all showed the same result. The redistribution in flow was sufficiently robust so that little change in flow occurred in vessels farther downstream from the occlusion.

A second test of the brain's vascular resilience involved a more traditional method to block flow that uses a fine filament threaded through the carotid to partially obstruct the middle cerebral artery, the major source of blood to the parietal cortex. While the flow is reduced throughout the entire surface network of communicating arterioles, a pattern of reversal in flows was also seen. Thus, reversals are a common feature in the redistribution of blood across the cortex.

Schaffer et al. show that the architecture of the cortical surface arterioles, with redundant connections between branches of the middle cerebral artery, ensures persistent blood flow and protects against localized occlusions. This extends the concept of redundant connections from a single loop in the circle of Willis, at the base of the brain, to the network of communicating arterioles on the cortical surface. Since humans and rats share a similar surface vasculature, these results could help identify potential links between vascular topology and stroke vulnerability in different regions of the brain.

